# Transcriptome and metabolome profiling reveal the chlorogenic acid as a resistance substance for rice against the white-backed planthopper *Sogatella furcifera* (Horváth)

**DOI:** 10.3389/fpls.2025.1571893

**Published:** 2025-05-30

**Authors:** Wenqi Xie, Wencai Tao, Huilai Zhang, Juntao Luo, Xiaoyue Deng, Deqiang Li, Qing Li, Haijian Wang, Yanli Yue, Surong Jiang, Chunxian Jiang

**Affiliations:** ^1^ College of Agronomy, Sichuan Agricultural University, Chengdu, China; ^2^ Sorghum Research Institute, Sichuan Academy of Agricultural Science, Deyang, China; ^3^ Emeishan Agricultural and Rural Bureau, Emeishan, China; ^4^ State Key Laboratory of Crop Gene Exploration and Utilization in Southwest China, Sichuan Agricultural University, Chengdu, China

**Keywords:** white-backed planthopper, rice, transcriptome, metabolome, chlorogenic acid, resistance

## Abstract

The white-backed planthopper (WBPH), *Sogatella furcifera* (Horváth) is a major migratory pest of rice, making research on rice resistance to WBPH essential for rice breeding and pest management. This study compared the resistance of susceptible rice TN1 and resistant rice KL35 to WBPH by analyzing antixenosis, antibiosis, and tolerance. We also conducted transcriptome and metabolome analysis to identify the defensive compounds against the WBPH and regulatory genes in KL35. The results indicated that KL35 exhibited significant antixenosis and tolerance to WBPH, markedly prolonging developmental duration and reducing fecundity. Metabolomic analysis identified 15 core metabolites, among which chlorogenic acid (CGA) content in KL35 was significantly higher than in TN1 both before and after WBPH feeding. Integrated transcriptomic and metabolomic analyses showed that the flavonoid biosynthetic pathway was a key anti-pest pathway in KL35. Additionally, two genes cinnamate 4-hydroxylase gene (*Os05g0320700*) and 4-coumarate CoA ligase (*Os02g0697400*) were identified and postulated as key players in the CGA biosynthesis pathway in KL35. Exogenous application of CGA to TN1 enhanced its tolerance and antixenosis to WBPH, significantly decreasing WBPH’s survival and mean dry weight. These findings suggest that CGA is an important resistance substance against WBPH. As a plant-derived and environment-friendly compound, CGA could be a potentially important compound for rice WBPH resistance agriculture.

## 1 Introduction

Rice (*Oryza sativa* L.) is one of the most important crops feeding more than half of the global population, and securing the production of rice is one of the critical activities for world food security, which is severely damaged by abiotic and biotic stresses, one of which is the white-backed planthopper (WBPH), *Sogatella furcifera* (Horváth) (Hemiptera: Delphacidae) (Divya et al., 2021; Ruan et al., 2021). The WBPH is one of the most destructive insect pests of rice in Asia (Li et al., 2016). WBPH feeds by penetrating plant cells and extracting large quantities of nutrients and moisture. When large numbers of WBPHs feed on rice, the leaves dry out, and the plant wilts, leading to the characteristic ‘hopper burn’ effect (Jang et al., 2021). More importantly, WBPH is a vector for devastating rice viruses, including the southern rice black-streaked dwarf virus (SRBSDV), further exacerbating the damage to rice crops (Matsukura et al., 2013). The long-distance migration and high fecundity of WBPH contribute to its outbreaks, which present significant challenges to rice production (Wang et al., 2017).

To overcome the challenge similar to WBPH damage to rice, researches had been conducted to reveal the plant defense systems to counter the insect herbivory and the secondary metabolites are tightly involved in those complex defense mechanisms by mediating different defensive functions and signaling pathways (War et al., 2012; Al-Khayri et al., 2023). Several studies have detected the effectiveness of the phenolic secondary metabolites against WBPH (Wang et al., 2020b; Jan et al., 2022), ferulic acid to brown planthopper (Yang et al., 2017), and tricin to BPH (Zhang et al., 2015). Also, the application of exogenous phenolics serves a highly similar effect as the endogenous ones through different functionalities (Movva and Pathipati, 2017; Chen et al., 2021).

Recent advances in metabolomics and transcriptomics have significantly enhanced our understanding of plant-insect interactions, providing a robust foundation for the application of these methods in this study (Zhang et al., 2022; Wang et al., 2023). Metabolome analysis, particularly differential accumulation of metabolites (DAMs), has been widely used to identify key secondary metabolites involved in plant defense mechanisms. For instance, in rice, metabolomic studies have revealed the role of phenolic compounds and flavonoids in conferring resistance against planthoppers (Wang et al., 2020c). Similarly, transcriptome analysis, focusing on differentially expressed genes (DEGs), has been instrumental in uncovering the genetic regulation of defense responses (Wang et al., 2020a; Sun et al., 2022)

Utilizing host plant resistance to breed insect-resistant rice varieties is an effective and economical strategy for controlling WBPH (Yang et al., 2014; He et al., 2015). Research on resistance genes for WBPH in rice has been ongoing for decades, leading to the development of resistant varieties (Romena et al., 1986; Yang and Zhang, 2016; Jan et al., 2022). With advances in molecular technologies, several resistance genes with significant effects against WBPH have been mapped in rice (Fan et al., 2018; Kim et al., 2021; Khan et al., 2022; Li et al., 2023). Research on rice resistance to the BPH has been far more extensive and in-depth compared to studies on resistance to the WBPH. The knowledge generated from BPH-resistant rice varieties—including the identification of resistance genes (e.g., Bph1 to Bph30), molecular mechanisms (e.g., salicylic acid/JA pathways), and advanced screening techniques—provides a robust foundation for accelerating WBPH resistance research (Yan et al., 2023; Ye et al., 2024).

The resistant rice KL35, developed through long-term selection, exhibits resistance to the WBPH, with moderate resistance during the mature stage and high resistance during the seedling stage. TN1 was one of the first semi-dwarf rice varieties, contributing to the breeding of high-yielding rice cultivars that revolutionized global rice production (Hargrove et al., 1979). Due to its high susceptibility to various pests and diseases, including the WBPH, TN1 has been widely used as a standard susceptible check in rice resistance studies (Li et al., 2019). Its genomic and phenotypic characteristics have been extensively analyzed, with recent chromosomal-level genome assembly providing valuable insights into its genetic composition and breeding potential (Panibe et al., 2021). Despite studies have focused on investigating the metabolic and molecular differences between planthopper-susceptible and resistant rice varieties following planthopper feeding (Li et al., 2022; Anand et al., 2023), comparative research focusing on the differential responses of KL35 and TN1 to WBPH infestation remains underexplored.

In this study, the susceptible rice TN1 was used as a control, and transcriptome and metabolome analyses were integrated to investigate the different mechanisms of KL35 on WBPH resistance. A secondary compound along with the associated genes in KL35 were detected and validated for rice WBPH resistance.

## 2 Materials and methods

### 2.1 Insects and plants

Two rice varieties were used in this study. TN1, a standard susceptible variety in resistance studies, has a well-characterized genome and was provided by the College of Agriculture of Sichuan Agricultural University. KL35, a resistant variety developed through long-term selection, shows moderate resistance to WBPH at maturity and high resistance at the seedling stage and was provided by the Rice and Sorghum Research Institute of the Sichuan Academy of Agricultural Science.

The WBPH strain was originally collected from a rice field in Luzhou, Sichuan, China in 2020 and maintained on the TN1. The 2nd-3rd instar nymphs and adults of the WBPH were selected for the study. The insects and rice plants were maintained in a climate chamber at 27 ± 1°C, with 70-80% relative humidity, and a light/dark photoperiod of 16/8 h.

### 2.2 Rice bioassays

#### 2.2.1 Antixenosis

Two-host choice test, slightly modified from the method of Zheng et al. (2021), was used to evaluate the antixenosis of rice varieties against WBPH nymphs and adults. Antixenosis against WBPH nymphs: seeds of KL35 and TN1 were sown in the same pot, with two plants of each variety planted 5 cm apart. At the two-leaf stage, 3 plants were retained per variety. Thirty 2nd-3rd instar nymphs were introduced and enclosed with a transparent cover. The treatment was repeated 10 times. Antixenosis against adult WBPH: seeds of KL35 and TN1 were sown in separate pots. At the two-leaf stage, 5 plants were retained per pot. Each insect-rearing cage contained one pot of KL35 and one pot of TN1, placed 5 cm apart. Ten pairs of newly emerged adult WBPH were released into each cage. The treatment was repeated 5 times. The number of WBPH nymphs and adults was recorded at 12, 24, 48, 72, and 96 h after insect release.

#### 2.2.2 Tolerance

Based on the method of Huang et al. (2016) with modifications, KL35 and TN1 were sown in separate pots. When the rice seedlings reached the two-leaf stage, 10 plants were retained per pot. Rice plants of the same variety were placed in an insect-rearing cage, with an average of 10 2nd-3rd instar nymphs introduced to each plant. Rice plants not fed with WBPH served as the control group. The treatment was repeated 10 times. The resistance level was assessed according to **Supplementary Table S1** (IRRI, 2002). Once the resistance level in any treatment group reached grade 9, rice samples were collected. Plants were cut at the soil level, washed, air-dried, and baked at 110°C for 30 min. They were then dried to a constant weight in a 60°C incubator and subsequently weighed. The functional plant loss index (FPLI) was calculated using the following formula.


$$\text{Functional plant loss index}\;\text{(FPLI})=100-\left(\frac{\text{Dry weight of infested plants}}{\text{Dry weight of uninfected plants}}\right)\times \left(1-\frac{\text{resistance level }}{9}\right)\times 100$$

#### 2.2.3 Antibiosis

Based on the method of Liu et al. (2022) KL35 and TN1 seedlings at the two-leaf stage were placed separately in cages containing adult WBPH for oviposition for 24 h. The seedlings were then removed, and the oviposition time was recorded before being transferred to rearing cages to await nymph hatching. First instar nymphs were collected for the treatment. Seedlings of KL35 and TN1 at the two-leaf stage were individually placed in tubes, with one seedling per tube. Each tube was inoculated with one newly hatched nymph sourced from the same rice varieties. The developmental status of the nymphs was observed and recorded daily, including molting and mortality. Each varieties was tested with 80 replicates. Seedlings were replaced as needed upon wilting. Male and female adults emerging on the same day from plants of the same variety were paired and transferred to fresh seedlings. Mortality was observed and recorded daily, with seedlings replaced as necessary, until all adults had died. The number of nymphs hatched on the seedlings was recorded daily. When no nymphs had hatched for 3 days, the seedlings were dissected under a stereomicroscope to count unhatched eggs. The total number of eggs laid was counted and the hatching rate was then calculated.

### 2.3 Sample preparation for metabolome and transcriptome analysis

KL35 and TN1 were sown in pots and placed separately in insect-rearing cages. Following the method of Zhang et al. (2022) at the two-leaf stage, an average of 10 2nd-3rd instar nymphs were introduced to each seedling. Samples were collected at 0 h and 48 h after feeding, resulting in four treatment groups: KL35_0 h, KL35_48 h, TN1_0 h, and TN1_48 h. Each treatment included 6 biological replicates for metabolomics and 3 biological replicates for transcriptomics. Rice seedlings from all treatment groups were sampled simultaneously, with 25 seedlings used per sample. Since WBPH primarily feeds on the stems of rice plants, the stems were mainly collected, while the leaves were not collected. The seedlings were washed with distilled water, and dried with filter paper, and the portion of the rice seedling surrounded by the first and second leaves was collected (which don’t include the leaves). The samples were immediately flash-frozen in liquid nitrogen and stored at -80°C for subsequent analysis.

### 2.4 Metabolome analyses

LC-MS/MS analysis was conducted using a UHPLC system (Thermo Fisher Scientific, USA) fitted with a Hypersil Gold column (2.1 mm × 100 mm, 1.9 µm). The aqueous mobile phase (phase A) consisted of water with 0.1% formic acid (positive ion mode) or 5 mmol/L ammonium acetate (negative ion mode), while the organic mobile phase (phase B) was methanol. The flow rate was set at 0.2 mL/min. The gradient elution was programmed as follows: 2% B for 1.5 min; 2-100% B from 1.5 to 12 min; 100% B from 12 to 14 min; 100-2% B from 14 to 14.1 min; and 2% B from 14.1 to 17 min. Raw data were preprocessed using Compound Discoverer 3.1 software. Metabolites identified in 24 samples were annotated through the KEGG (https://www.genome.jp/kegg/pathway.html), HMDB (https://hmdb.ca/metabolites), and LIPID MAPS databases (http://www.lipidmaps.org/) (Aoki and Kanehisa, 2005; Sud et al., 2007; Wishart et al., 2009). Data processing was performed using the MetaX software, followed by multivariate statistical analysis of the processed data through Principal Component Analysis (PCA) and Partial Least Squares-Discriminant Analysis (PLS-DA) (Boulesteix and Strimmer, 2006; Wen et al., 2017). The DAMs were identified by variable importance in projection (VIP) >1 and fold change (FC) ≥1.2 or FC ≤0.833. BeijingNovogene Co. Ltd (Beijing, China) was employed for all metabolomic analyses.

### 2.5 Transcriptome analysis

RNA Extraction from samples using the RNAprep Pure Plant Plus Kit (Polysaccharides & Polyphenolics-rich) (TIANGEN, Beijing, China) and then the qualified RNAs were used for transcriptome sequencing using the Illumina platform (Novogene, Beijing, China). The clean data (clean reads) were obtained by removing reads with adapters, removing reads containing N (where N represents undetermined base information), and removing low-quality reads (reads where the number of bases with Qphred ≤ 20 accounts for more than 50% of the total read length). Raw paired-end reads were cleaned with fastp (Chen et al., 2018) and cleaned reads were aligned to the indexed reference genome Nipponbare IRGSP-1.0 (Shi et al., 2023) with Hisat2 v2.0.5 (Kim et al., 2015). The mapped reads of each sample were assembled by StringTie (vl.3.3b) in a reference-based approach (Pertea et al., 2015). FeatureCount v1.5.0-p3 (Liao et al., 2014) was used to count the mapped read numbers by gene; And then FPKM (Conesa et al., 2016) of each gene was calculated based on the length of the gene and reads countmapped to this gene. The differential expression analysis was performed using DESeq2 (Love et al., 2014), and genes were considered as DEGs if padj ≤0.05 and |log_2_(FoldChange)| >1. Functional enrichment analysis was performed to identify DEGs that were significantly enriched in Gene Ontology (GO, http://geneontology.org/) terms and Kyoto Encyclopedia of Gene and Genomes (KEGG, http://www.genome.jp/kegg/) pathways (Kanehisa et al., 2007; The Gene Ontology Consortium, 2019). BeijingNovogene Co. Ltd (Beijing, China) was employed for all transcriptome analyses.

### 2.6 Combined transcriptomic and metabolomic analysis

To investigate the correlation between DEGs and DAMs, we conducted a correlation analysis of the transcriptome and metabolome. Overlapping pathways between the transcriptome and metabolome were identified, and the corresponding KEGG files were downloaded from the KEGG pathway database. The key DEGs and metabolic pathways between KL35 and TN1 were identified and presented.

### 2.7 Quantitative reverse-transcription polymerase chain reaction

Based on the integrated analysis of metabolomic and transcriptomic data, the cinnamate 4-hydroxylase gene (C4H, *Os05g0320700*) and 4-coumarate CoA ligase (4CL, *Os02g0697400*), both involved in the regulation of the chlorogenic acid (CGA) synthesis pathway, were identified and validated through qRT-PCR. Specific primers (80–150 bp) for each gene were designed using primer premier 5.0, based on the coding sequences (CDS) of the genes queried on NCBI (**Supplementary Table S2**). *Actin 11* (AK1002267) was used as the internal control (Primerbank, 2017). Total RNA was extracted from rice using FastPure Plant Total RNA Isolation Kit (Vazyme, Nanjing, Jiangsu, China). The extracted RNA was quantified using a NanoDrop 2000 spectrophotometer, and its purity was evaluated via 1% agarose gel electrophoresis. First-strand cDNA was synthesized using HiScript^®^ II Q RT Super Mix for Qpcr (+gDNA wiper) (Vazyme, Nanjing, Jiangsu, China). ChamQ Universal SYBR qPCR Master Mix (Vazyme, Nanjing, Jiangsu, China) was used in qRT-PCR. Relative expression data were analyzed using the 2^-△△ct^ method (Livak and Schmittgen, 2001). All qRT-PCR experiments used four technical replicates and three biological replicates.

### 2.8 Effects of exogenous CGA on WBPH bioassays

Determination of WBPH survival rate and average dry weight: TN1 was sown in hydroponic cassettes, and exogenous CGA was applied at the two-leaf stage of rice seedlings. CGA solutions were prepared at concentrations of 50 μM and 200 μM, with distilled water used as a control. The solutions were sprayed onto the rice plants. One hour after treatment, 3 rice seedlings were placed in a single glass tube, and 30 2nd-3rd instar nymphs were introduced, with a control group set up without insect introduction. Each treatment was repeated 6 times. The number of surviving WBPH was recorded at 24 h, 48 h, 72 h, and 96 h, and the survival rate was calculated. At 96 h, the total number of WBPH (including dead ones) in each glass tube was counted. The WBPHs were then dried in an oven for 4 h, and the dry weight was recorded to calculate the average dry weight.

Antixenosis: Refer to section 2.2.1. TN1 was sown in two rows in the same pots, maintaining a 5 cm spacing between the rows. At the two-leaf stage, 3 plants were retained per row. A 200μM CGA solution was sprayed on one row of the rice seedlings, and an equivalent amount of distilled water was sprayed on the other row as control. One hour after treatment, 30 2nd-3rd instar nymphs were introduced between the two rows and enclosed with a transparent cover. The number of WBPH nymphs was recorded on each row of rice seedlings at 12, 24, 48, and 72 h post-feeding. Each treatment was conducted in 4 replicates.

Tolerance: Refer to section 2.2.2. TN1 was sown in pots placed inside insect-rearing cages. When the rice seedlings reached the two-leaf stage, 10 seedlings per pot were retained. A 200 μM CGA solution was prepared and sprayed onto the rice plants, while an equivalent amount of distilled water was applied as a control. One hour after treatment, each seedling was inoculated with an average of 10 2nd-3rd instar nymphs. An additional group was set as a non-feeding control, where seedlings were not exposed to WBPH nymphs but were otherwise treated identically. Subsequent sample collection, processing, and result evaluation were performed following the procedures outlined in Section 2.2.2.

### 2.9 Statistical analysis

The experimental data were tested for normality and homogeneity of variances using the Shapiro-Wilk and Levene tests in SPSS 27 software (Ghasemi and Zahediasl, 2012; Pallant, 2020). A t-test was used to analyze the significant differences in antixenosis, tolerance, hatching rate, feeding preference, the FPLI, and changes in plant dry weight before and after WBPH feeding. One-way analysis of variance (ANOVA) with the LSD method was employed to compare significant differences in the survival rate and average dry weight of WBPH. For antibiosis assessment, the data were analyzed using the TWOSEX-MSChart software for two-sex life table analysis (Chi, 1988). The parameters calculated included the developmental duration, lifespan, survival rate, adult pre-oviposition period (APOP), total pre-oviposition period (TPOP), intrinsic rate of increase (*r*), finite rate of increase (λ), net reproductive rate (*R*_0_), and mean generation time (*T*) for WBPH reared on different rice varieties. The bootstrap technique included in TWOSEX-MSChart was used to estimate the means and standard errors of life table parameters, based on 100,000 random resampling iterations (Chi et al., 2022). Differences in life table parameters were assessed using the paired bootstrap test.

## 3 Results

### 3.1 Rice bioassays

#### 3.1.1 The antixenosis of KL35 to WBPH

**Figure 1:**
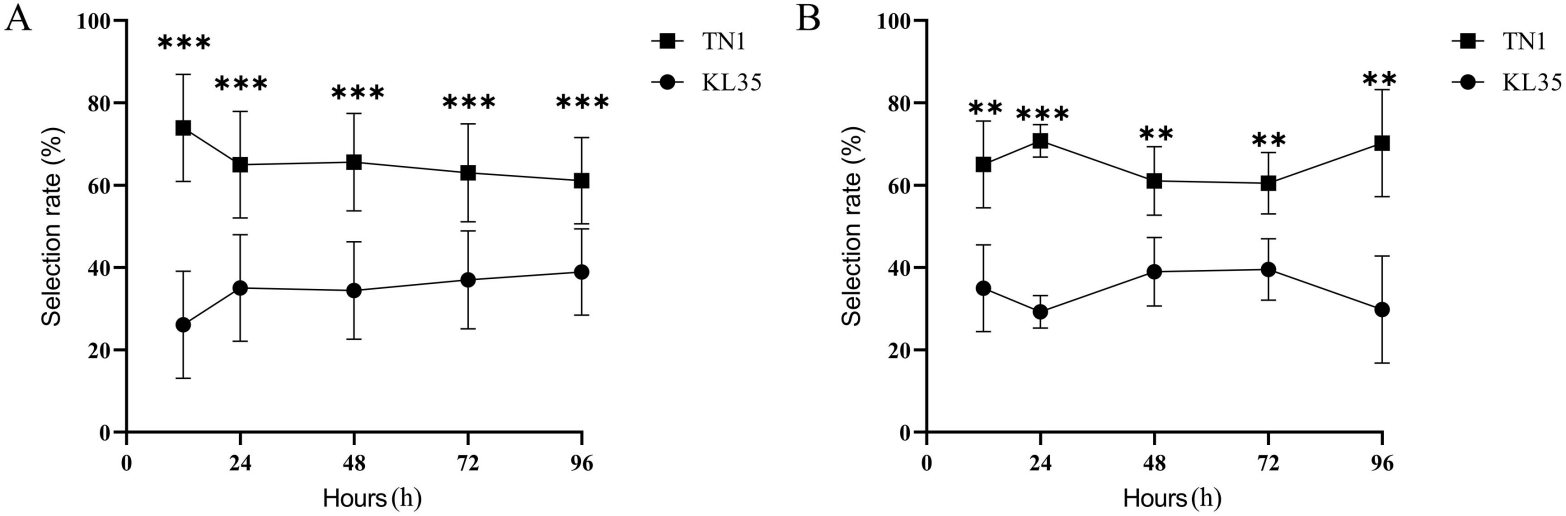
Comparison of WBPH selection profiles between KL35 and TN1. **(A)** nymphs, **(B)** adults. **, and *** represent significance p-level of 0.01, and 0.001, respectively, for differences with independent sample t-test.

The results presented in **Figure 1** demonstrated distinct differences in the selection rates of WBPH nymphs and adults between the KL35 and the TN1. The selection rate for WBPH nymphs on KL35 was significantly lower than that on TN1 at all time points after feeding. After 12 hours of feeding, the selection rate on KL35 was the lowest, at 26.10%, which showed the presence of an antixenotic effect. With prolonged observation, the selection rate gradually increased before stabilizing, potentially reflecting a partial adaptation of the insects to the resistant variety, though the initial deterrent effect seemed to persist to some extent. Similar results were observed in the antixenosis test with adult WBPH. At all the time points, the selection rate for KL35 was significantly lower than that for TN1. At 24 h, the selection rate of WBPH adult for KL35 decreased further compared to 12 h, reaching the lowest value of 29.81% at 96 h.

#### 3.1.2 The tolerance of KL35 to WBPH

As shown in **Table 1**, the dry weight of TN1 showed a notable reduction following WBPH feeding, with the loss being more pronounced compared to that of KL35. This showed that TN1 was more susceptible to damage caused by WBPH infestation. Furthermore, The FPLI of TN1 was significantly higher than that of KL35, which implied greater feeding activity or damage on TN1. Additionally, the total dry weight of WBPH feeding on KL35 was significantly lower compared to those feeding on TN1 provided additional confirmation for the experimental results mentioned above.

**Table 1:** Evaluation of tolerance to WBPH feeding among rice varieties.

Varieties	Total dry weight of uninfected plants (g)	Total dry weight of infested plants (g)	The average resistance level at sampling time	Total dry weight of WBPH	Functional plant loss index (FPLI)
TN1	0.43 ± 0.02^ns^	0.28 ± 0.01	8.20 ± 0.50^**^	20.84 ± 1.09^**^	94.73 ± 3.28^**^
KL35	0.40 ± 0.02	0.33 ± 0.01^*^	3.40 ± 0.75	16.70 ± 0.43	48.51 ± 7.06

**, *, and ns indicate a significant difference (*p*< 0.01), a significant difference (*p*< 0.05), and no significant difference (*p* > 0.05) determined by independent samples’ t-test.

#### 3.1.3 The antibiosis of KL35 to WBPH

Compared with TN1, KL35 displayed prolonged development duration and significant inhibited reproduction of WBPH (**Table 2**). The egg stage, pre-adult stage, APOP and TPOP of WBPH on KL35 were significantly longer than those on TN1. Additionally, the oviposition quantity per female and the hatching rate of WBPH on KL35 were notably lower than on TN1. From the population parameters of WBPH feeding on different rice varieties (**Table 3**), it was observed that the intrinsic rate of increase (*r*, 0.143), finite rate of increase (*λ*, 1.154), and net reproductive rate (*R_0_*, 44.20) of WBPH feeding on KL35 were significantly lower than those feeding on TN1.

**Table 2:** The influence between TN1 and KL35 affected the development duration, longevity, and reproductive parameters of WBPH.

Stage	TN1	KL35
Egg (d)	6.64 ± 0.05 (80)	6.88 ± 0.04^*^ (80)
1st-instar nymph (d)	2.67 ± 0.05^ns^ (78)	2.67 ± 0.05 (75)
2nd-instar nymph (d)	1.59 ± 0.06 (78)	1.69 ± 0.04^ns^ (74)
3rd-instar nymph (d)	1.88 ± 0.05 (78)	2.01 ± 0.07^ns^ (73)
4th-instar nymph (d)	2.05 ± 0.05^ns^ (77)	1.99 ± 0.05 (72)
5th-instar nymph (d)	2.74 ± 0.06^ns^ (77)	2.74 ± 0.06 (70)
Pre-adult duration (d)	17.55 ± 0.15 (77)	18.01 ± 0.09^*^ (70)
Adult duration (d)	12.22 ± 0.55 (77)	12.50 ± 0.536^ns^ (70)
Female longevity (d)	32.17 ± 0.63^ns^ (34)	31.37 ± 0.72 (32)
Male longevity (d)	27.88 ± 0.80 (43)	29.79 ± 0.79^ns^ (38)
APOP (d)	2.03 ± 0.25 (34)	2.84 ± 0.24^*^ (32)
TPOP (d)	19.86 ± 0.28 (34)	20.91 ± 0.27^*^ (32)
Oviposition days (d)	10.56 ± 0.56^ns^ (34)	8.91 ± 0.67 (32)
Fecundity (offspring/individual)	162.82 ± 10.72^*^ (34)	110.5 ± 10.60 (32)
Hatching rate (%)	85.82 ± 2.02^*^ (34)	66.57 ± 2.01 (32)

The values in the table are presented as MEAN ± SE. * and ns indicate a significant difference (*p*< 0.05), and no significant difference (*p* > 0.05) determined by independent samples’ t-test in the hatching rate and fecundity (offspring/individual). * and ns indicate a significant difference (*p*< 0.05), and no significant difference (*p* > 0.05) determined by TWOSEX-MSChart software for two-sex life table analysis in the rest of the data.

**Table 3:** The influence between TN1 and KL35 affected on the population parameters of WBPH.

Population parameters	TN1	KL35
The intrinsic rate of increase, *r* (d^-1^)	0.162 ± 0.006^*^	0.143 ± 0.00
Finite rate of increase, *λ* (d^-1^)	1.177 ± 0.007^*^	1.154 ± 0.007
Net reproductive rate, *R_0_* (eggs/individual)	69.20 ± 10.06^*^	44.20 ± 7.41
Mean generation time, *T* (d)	26.022 ± 0.257	26.458 ± 0.332^ns^

The values in the table are presented as MEAN ± SE. * and ns indicate a significant difference (*p*< 0.05), and no significant difference (*p* > 0.05) determined by TWOSEX-MSChart software for two-sex life table analysis

### 3.2 Metabolome analyses

**Figure 2:**
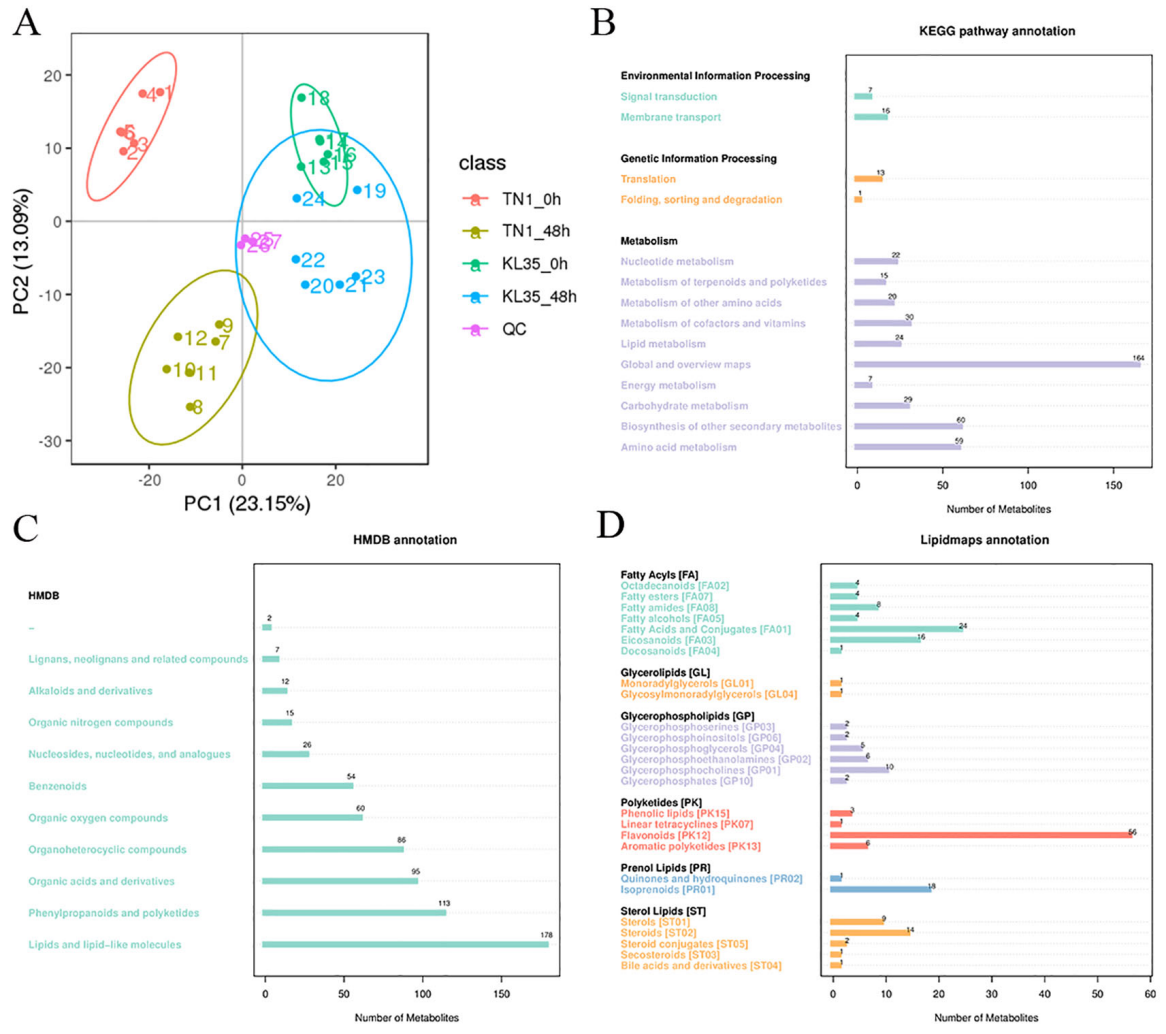
Annotation of metabolites profiling with different databases. **(A)** Principal component analysis of all detected metabolites in both TN1 and KL35; **(B)** KEGG; **(C)** HDMB, **(D)** LIPID MAPS.

Principal component analysis (PCA) indicated that all QC samples clustered tightly together, demonstrating good reproducibility and reliable data (**Figure 2A**). A total of 1,276 metabolites were detected in this study (**Supplementary Table S3**). According to the KEGG database annotation, 23 metabolites were assigned to the environmental information processing pathway, 14 metabolites to the genetic information processing pathway, and 430 metabolites to the metabolic pathway (**Figure 2B**). Annotation results from the HMDB database showed that the largest categories of metabolites were lipids and lipid-like molecules (178), phenylpropanoids and polyketides (113), and organic acids and derivatives (95) (**Figure 2C**). In the LIPIDMAPS database annotation, the most abundant metabolites were polyketides (66), fatty acyls (61), glycerophospholipids (27), and sterol lipids (27) (**Figure 2D**).

A total of 314 DAMs were identified in TN1 rice before and after WBPH feeding (**Supplementary Table S4**), while 198 DAMs were identified in KL35 (**Supplementary Table S5**). Additionally, 793 DAMs were identified between the TN1 and KL35 (**Supplementary Table S6**). Among these, several phenolic compounds were detected, including phenolic acids such as caffeic acid, p-coumaric acid, ferulic acid, cinnamic acid, and chlorogenic acid, as well as flavonoids such as albiflorin, eriodictyol, luteolin, apigenin, quercetin, hesperetin, naringenin, isorhamnetin, and myricetin.

**Figure 3:**
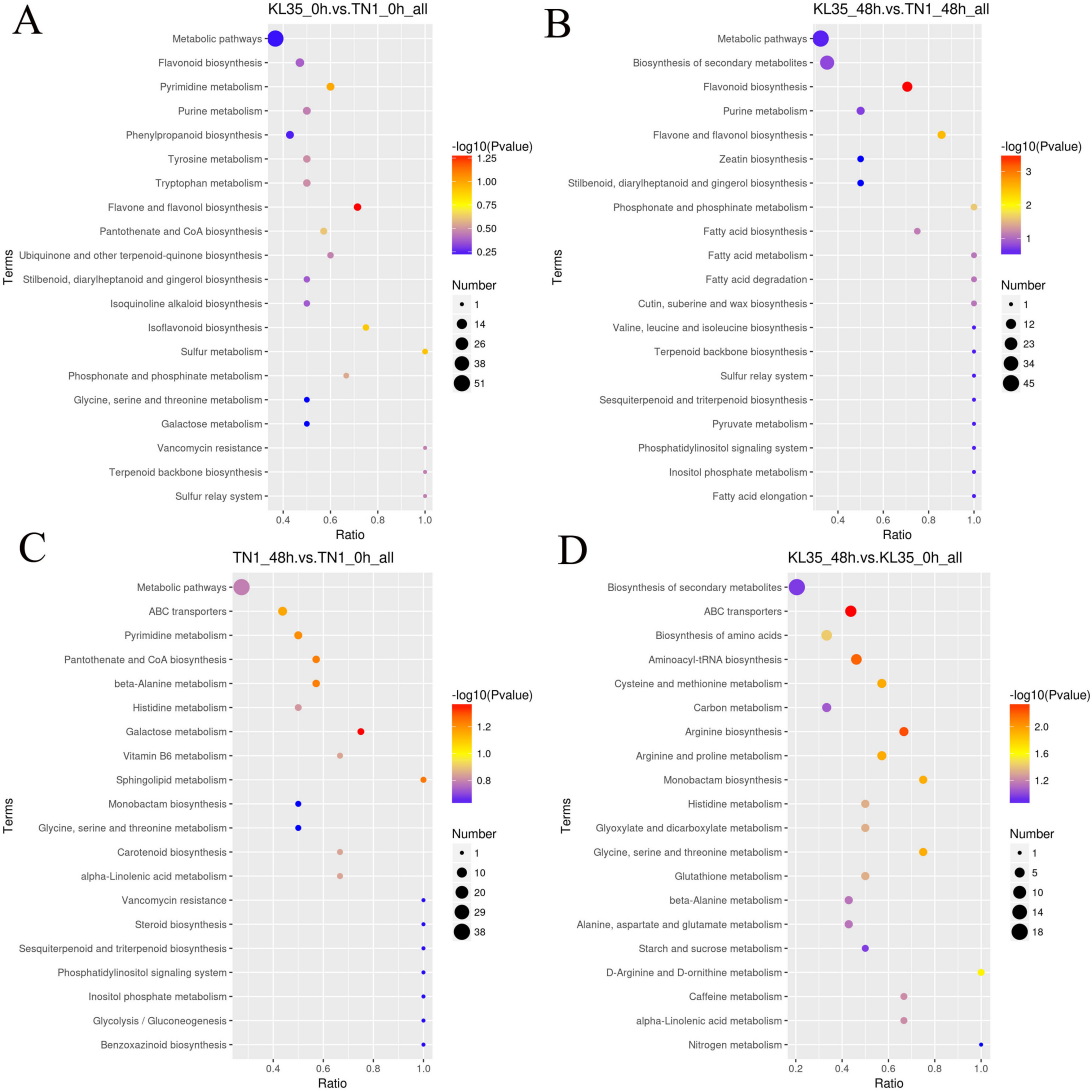
KEGG pathway analysis of differentiated DAMs between 2 lines and between 2 time points. **(A)** KL35 vs TN1 at 0h; **(B)** KL35 vs TN1 at 48h; **(C)** 48h vs 0h for TN1; **(D)** 48h vs 0h for KL35.

The DAMs in KL35_0h vs TN1_0h were enriched in 32 metabolic pathways, including phenylalanine metabolism, flavone, and flavonol biosynthesis, and flavonoid biosynthesis (**Figure 3A**). The DAMs in KL35_48h vs TN1_48h were enriched in 39 metabolic pathways, with significant enrichment in 3 pathways: flavonoid biosynthesis, flavone and flavonol biosynthesis, and phosphonate and phosphinate metabolism (*p*< 0.05) (**Figure 3B**). The DAMs in TN1_48h vs TN1_0h were enriched in 44 metabolic pathways, with significant enrichment in the galactose metabolism pathway (*p*< 0.05) (**Figure 3C**). The DAMs in KL35_48h vs KL35_0h were enriched in 48 metabolic pathways, including plant hormone signal transduction, biosynthesis of secondary metabolites, and phenylalanine metabolism, with significant enrichment in 12 pathways, such as ABC transporters, Arginine biosynthesis, and Aminoacyl-tRNA biosynthesis (*p*< 0.05) (**Figure 3D**). All differentiated metabolites between TN1 and KL35 are listed in **Supplementary Table S7**. Both TN1 and KL35 shared core metabolic pathways, particularly in phenylalanine metabolism flavone and flavonol biosynthesis, and flavonoid biosynthesis, at both time points. Over time, TN1 and KL35 exhibited divergent metabolic strategies. TN1 showed a focused adaptation in galactose metabolism, while KL35 underwent a broader and more complex metabolic reprogramming, involving plant hormone signaling, secondary metabolite biosynthesis, and amino acid metabolism. KL35 demonstrated a more dynamic and extensive metabolic response over time compared to TN1, which undergoes more specific changes.

**Figure 4:**
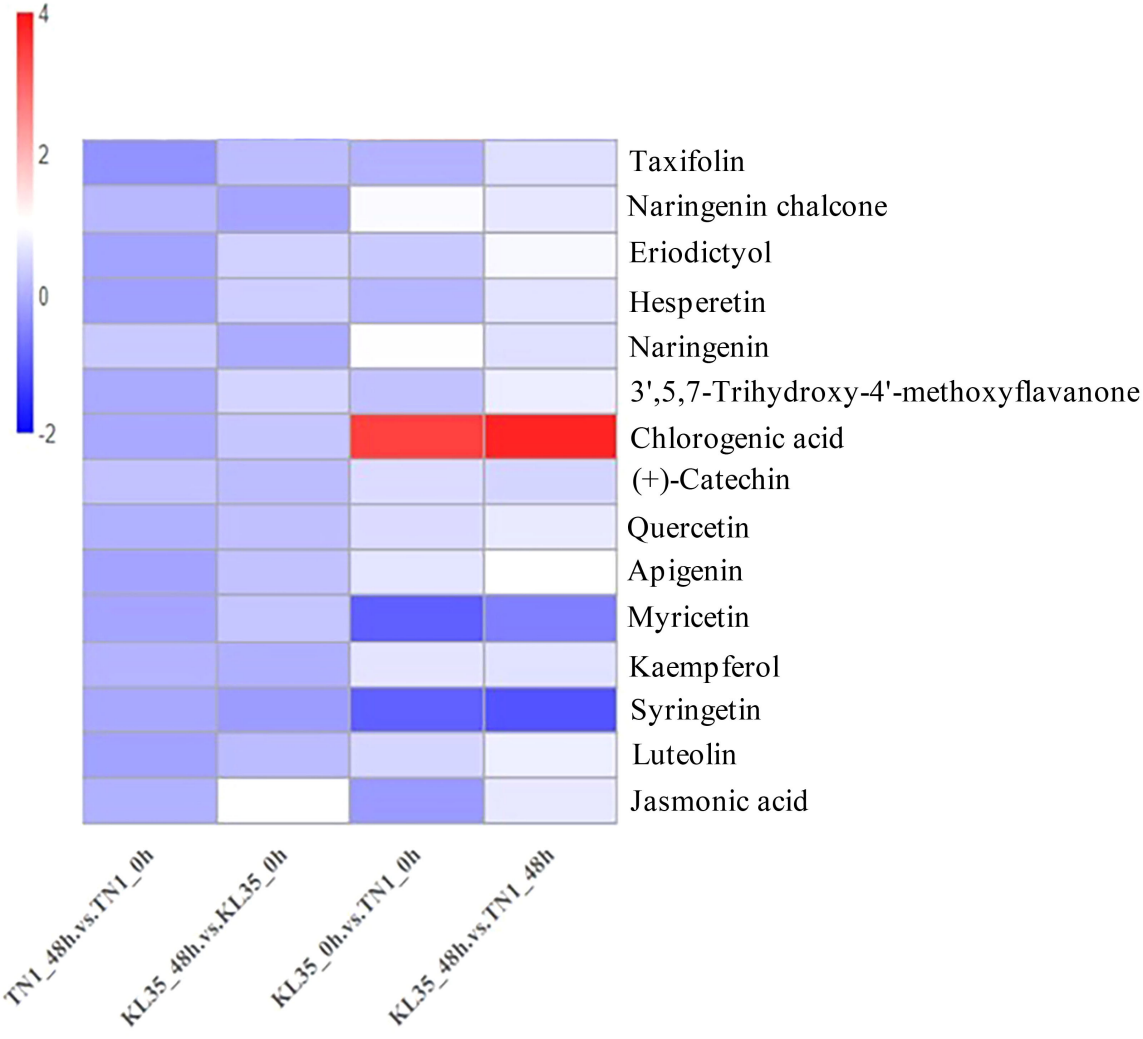
Heatmap of changes in core metabolites between KL35 and TN1. The x-axis represents the various treatment groups, while the y-axis represents the names of the metabolites, plotted according to their log_2_ fold change (log_2_FC) values.

Based on flavone and flavonol biosynthesis, flavonoid biosynthesis, and plant hormone signal transduction, this study identified 15 key metabolites (**Figure 4**; **Supplementary Table S8**). From the flavonoid biosynthesis pathway and the flavone and flavonol biosynthesis pathway, the following metabolites were identified: taxifolin, naringenin chalcone, eriodictyol, hesperetin, naringenin, (+)-catechin, 3’,5,7-trihydroxy-4’-methoxyflavanone, chlorogenic acid (CGA), quercetin, apigenin, myricetin, kaempferol, diosmetin, and luteolin. From the plant hormone signal transduction pathway, jasmonic acid (JA) was identified and was significantly upregulated in KL35 under WBPH feeding induction. Among these metabolites, CGA showed significant differences between KL35 and TN1. Before feeding, the CGA content in KL35 was 11.08 times higher than in TN1, and after feeding, it was 14.07 times higher. Additionally, the CGA content in KL35 showed an increasing trend after WBPH feeding.

### 3.3 Transcriptome analyses

**Figure 5:**
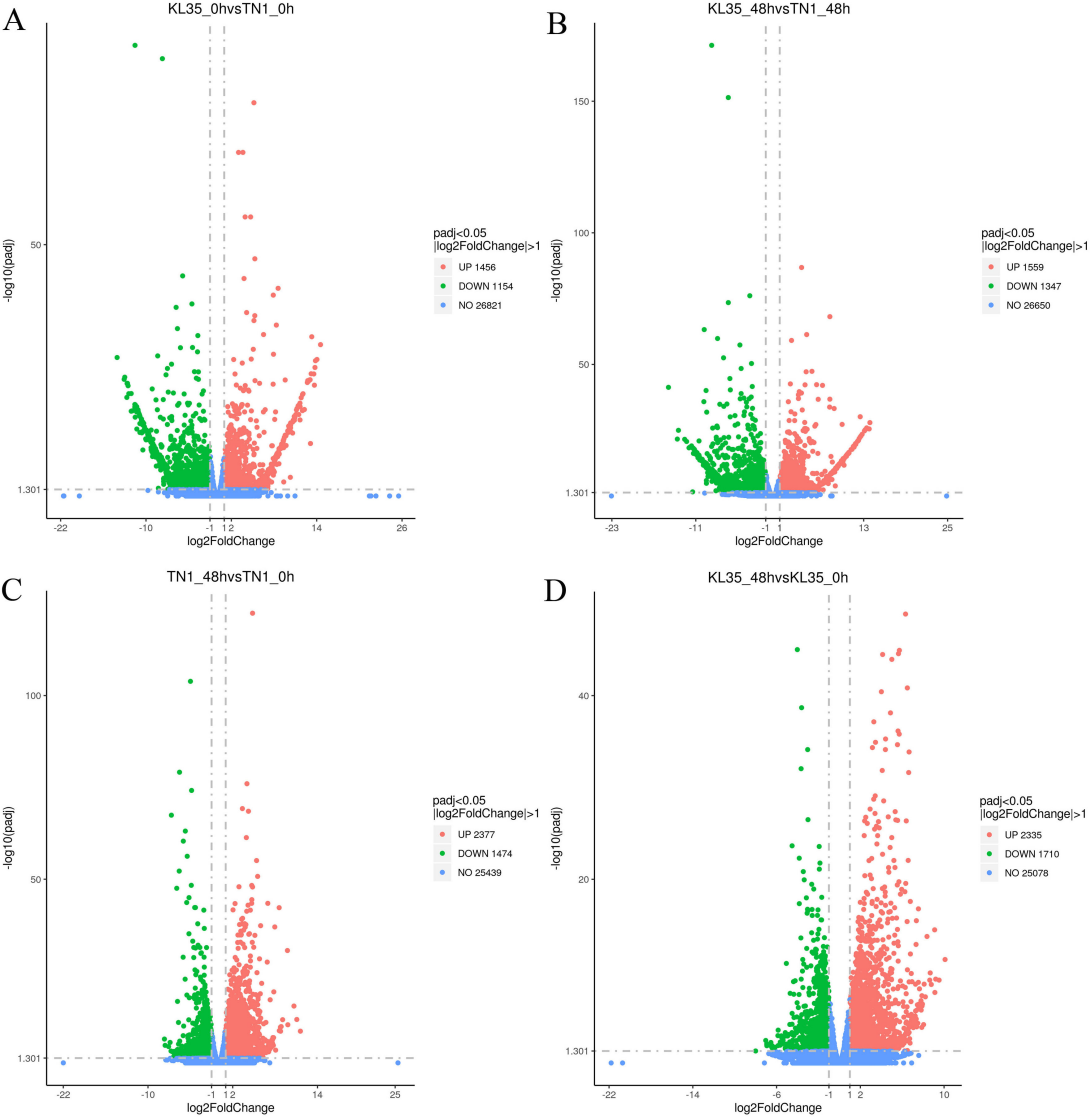
Volcano plots of DEGs between 2 lines and between 2 time points. **(A)** KL35 vs TN1 at 0h; **(B)** KL35 vs TN1 at 48h; **(C)** 48h vs 0h for TN1; **(D)** 48h vs 0h for KL35, with |fold change|> 1 with padj ≤ 0.05. Up/down-regulated and no change genes are represented by red, green, and blue, respectively.

The DEGs and their upregulation or downregulation in TN1 and KL35, as well as between the susceptible and resistant rice varieties before and after WBPH feeding, are shown in **Figure 5**. In TN1 after WBPH feeding, 3851 DEGs were identified, including 2,377 upregulated and 1,474 downregulated genes (**Supplementary Table S9**). In KL35, 4,045 DEGs were identified, with 2,335 upregulated and 1,710 downregulated genes. There were also differences in the total number of DEGs and their regulation patterns between the susceptible and resistant varieties before and after feeding (**Supplementary Table S10**). These results indicated that TN1 and KL35 exhibited different gene expression patterns in response to WBPH stress. KL35 likely contains key resistance genes that regulate relevant defense pathways, enabling it to withstand WBPH stress.

**Figure 6:**
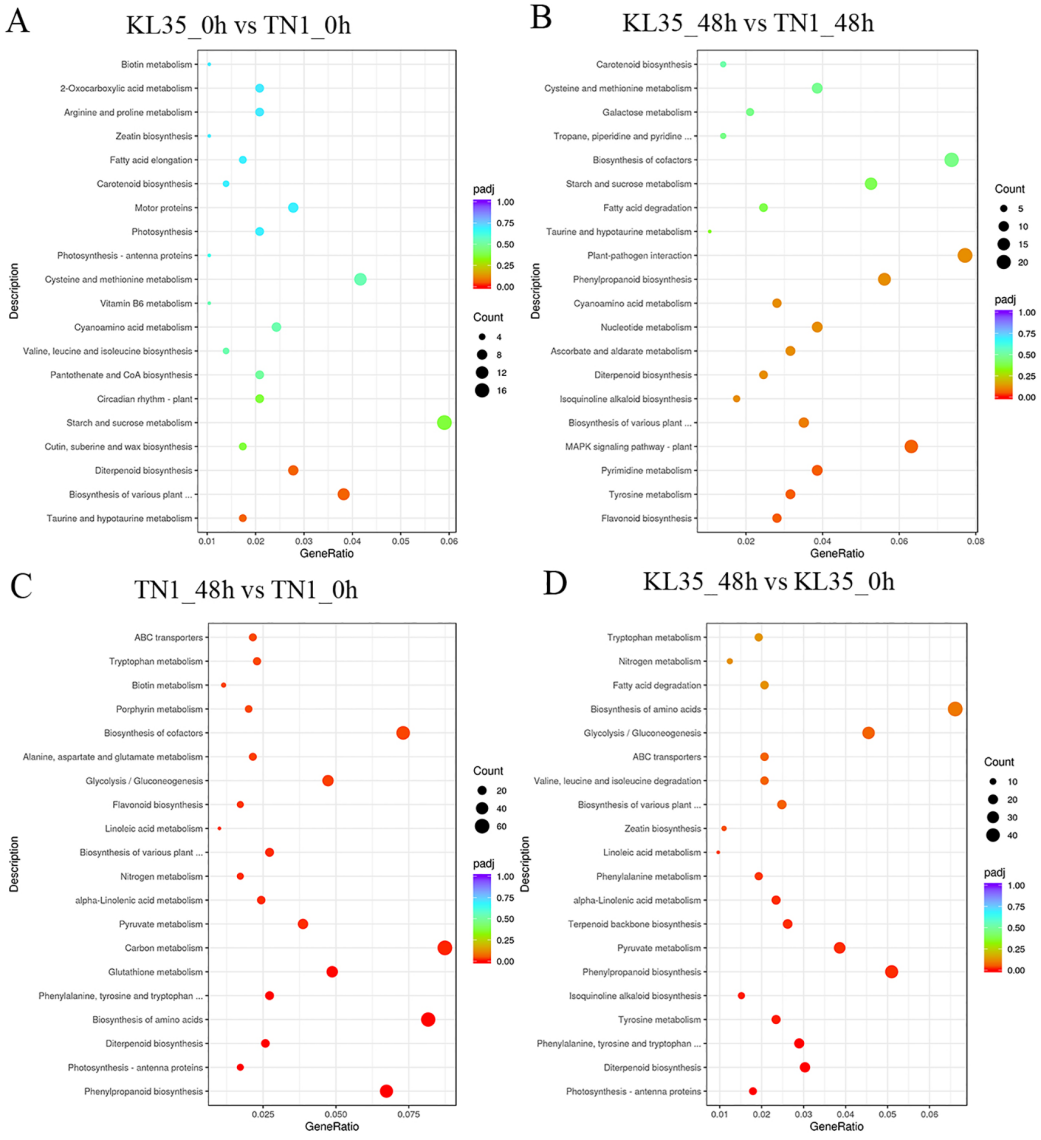
KEGG analysis within between lines for 2 time points **(A, B)** and each line at different time points **(C, D)**. Enriched DEGs numbers are represented by circle size, and significance levels by color. **(A)** KL35 vs TN1 at 0h; **(B)** KL35 vs TN1 at 48h; **(C)** 48h vs 0h for TN1; **(D)** 48h vs 0h for KL35.

The DEGs in KL35_0h vs TN1_0h were significantly enriched in 10 pathways, including taurine and hypotaurine metabolism, biosynthesis of various plant secondary metabolites, and diterpenoid biosynthesis (*p*< 0.05) (**Figure 6A**). This showed that TN1 undergoes extensive metabolic changes over time, with a strong focus on primary metabolism and stress-related secondary metabolite biosynthesis. The DEGs in KL35_48h vs KL35_0h were significantly enriched in 23 pathways, such as phenylalanine metabolism, biosynthesis of various plant secondary metabolites, and photosynthesis - antenna proteins (*p*< 0.05) (**Figure 6B**). While KL35 also showed changes in primary and secondary metabolism, the number of enriched pathways is smaller compared to TN1, suggesting a more targeted response to WBPH stress. The DEGs in TN1_48h vs TN1_0h were significantly enriched in 39 pathways, including phenylalanine metabolism, photosynthesis - antenna proteins, and diterpenoid biosynthesis (*p*< 0.05) (**Figure 6C**). This indicated that, even at the initial time point, the two lines exhibited differences in secondary metabolite biosynthesis and stress-related pathways, suggesting distinct baseline metabolic states. The DEGs in KL35_48h vs TN1_48h were significantly enriched in 14 pathways, such as flavonoid biosynthesis, biosynthesis of various plant secondary metabolites, and the MAPK signaling pathway - plant (*p*< 0.05) (**Figure 6D**). This revealed that, under WBPH stress, both lines shared some common responses, particularly in secondary metabolite biosynthesis and stress signaling, but the specific pathways and degrees of enrichment differ. TN1 prioritized primary metabolism and energy-related processes, whereas KL35 employed a more balanced approach, integrating primary and secondary metabolism with stress signaling.

### 3.4 Integrated analyses of the transcriptomic and metabolic data

**Figure 7:**
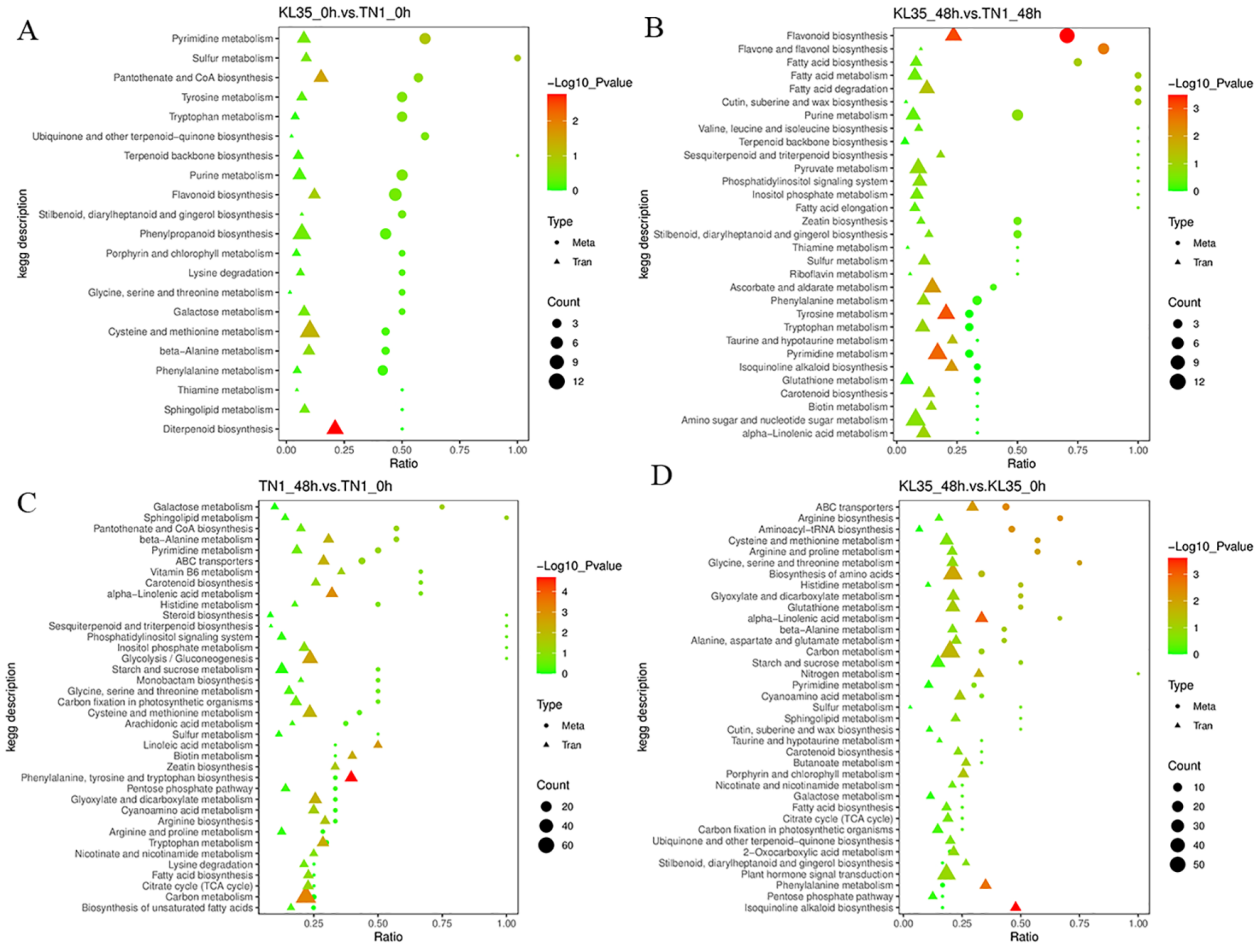
Integration of DAMs and DEGs from KEGG pathway analysis between 2 lines and between 2 time points. **(A)** KL35 vs TN1 at 0h; **(B)** KL35 vs TN1 at 48h; **(C)** 48h vs 0h for TN1; **(D)** 48h vs 0h for KL35. Metabolic pathways are presented along the y-axis, and the enrichment ratio by the x-axis. Analysis categories are indicated by symbol shapes. Circle for metabolomic analysis, and triangle, transcriptomic analysis. The shape size corresponds to the numbers of DEGs or DAMs with color for enrichment levels as green, yellow, and red indicating low, moderate, and high, respectively. Heatmaps were generated using log2-normalized FPKM values.

The KEGG pathway enrichment analysis was performed on DAMs and DEGs to identify the key biochemical and signaling pathways jointly involved by these factors (**Figure 7**; **Supplementary Table S11**). Both DEGs and DAMs between KL35 and TN1 were significantly enriched in the flavonoid biosynthesis pathway (*p*< 0.05). The DAMs enriched in the flavonoid biosynthesis pathway between susceptible and resistant varieties included taxifolin, naringenin chalcone, quercetin, myricetin, eriodictyol, hesperetin, kaempferol, naringenin, 3’,5,7-trihydroxy-4’-methoxyflavanone, luteolin, chlorogenic acid, and apigenin. The DEGs included *Os12g0115700*, *Os03g0819600*, *novel.1556*, *Os08g0498600*, *Os05g0320700*, *Os04g0630600*, *Os04g0630100*, *Os04g0630800*, and *Os03g0367101*. The flavonoid biosynthesis pathway is a secondary metabolic pathway in plants that plays an important role in plant defense, helping to protect against biotic stress. The significant enrichment of DAMs and DEGs in this pathway between the susceptible and resistant varieties indicated a strong correlation between the insect resistance mechanism of KL35 and this pathway.

### 3.5 Analysis of CGA synthesis regulatory pathway and qRT-PCR validation of related genes

**Figure 8:**
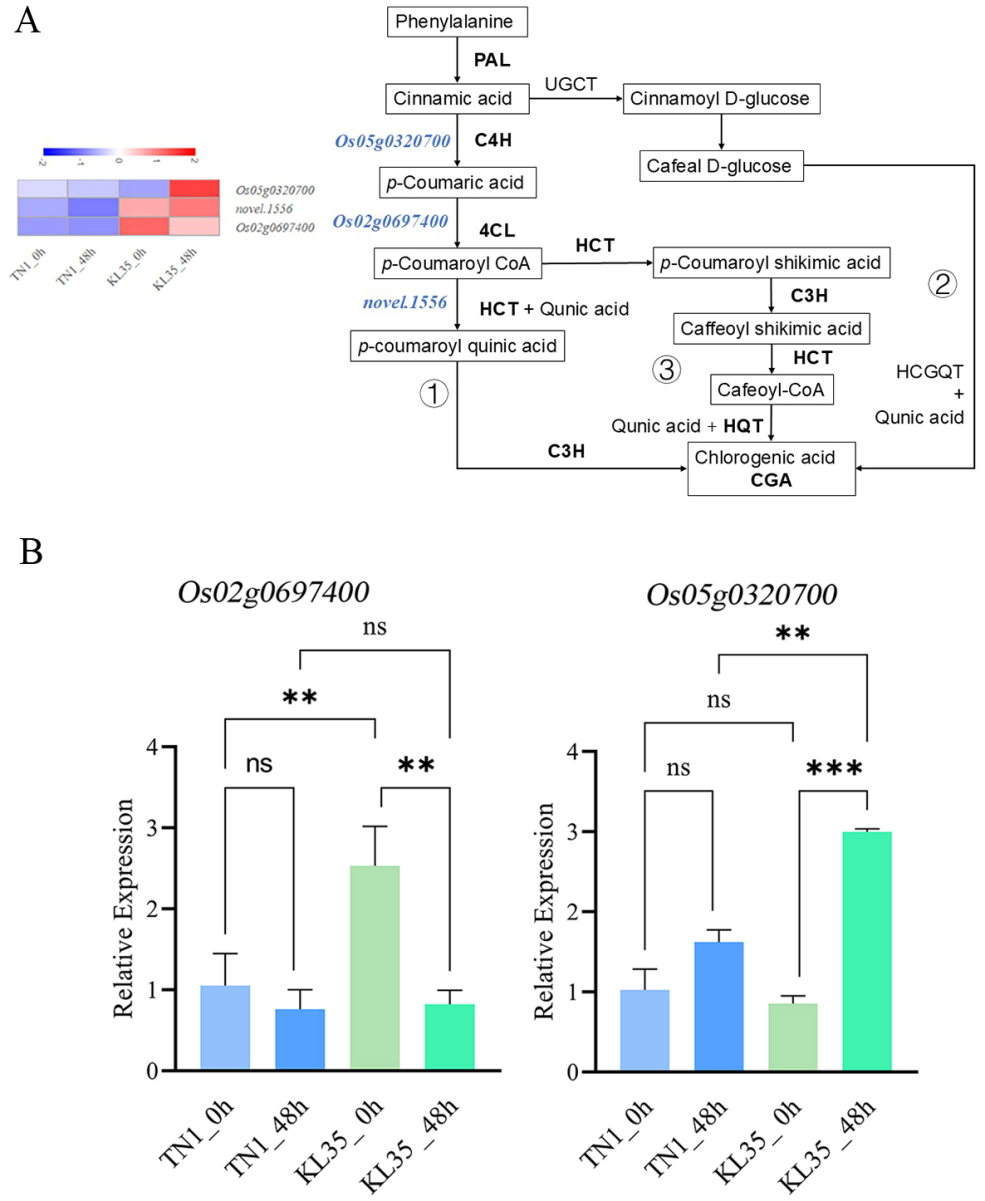
**(A)** Diagram of CGA biosynthesis pathway and expression of selected genes with RT-qPCR PAL, phenylalanine ammonia-lyase; C4H, cinnamate 4-hydroxylase; 4CL, 4-coumarate CoA ligase; HCT, hydroxycinnamoy-CoA shikimate/quinate hydroxycinnamoyl transferase; C3H, coumarate 3-hydroxylase; HQT, hydroxycinnamoyl-CoA quinate hydroxycinnamoyl transferase; UGCT, UDP-glucose, cinnamate glucosyltransferase; HCGQT, hydroxycinnamoyl-glucose, quinate hydroxycinnamoyltransferase. The heatmap at the left is the gene expression profile. **(B)** Quantitative results of RT-qPCR for CGA synthesis regulatory genes (*Os02g0697400*, *Os05g0320700*). ***, **, and ns indicate significance levels of p< 0.001, 0.01, and no significance, respectively, with independent samples t-test.

Transcriptome and integrated analyses revealed that, regardless of whether WBPH was feeding, the difference in CGA content between TN1 and KL35 was the most significant among the differential metabolites. Therefore, the CGA synthesis regulatory pathway was analyzed to investigate the insect resistance mechanism of KL35 (**Figure 8A**). In the KL35_0h vs TN1_0h and KL35_48h vs TN1_48h comparison groups, the genes encoding C4H (*Os05g0320700*), 4CL (*Os02g0697400*), and hydroxycinnamoyl-CoA shikimate/quinate hydroxycinnamoyl transferase gene (HCT, *novel.1556*) were significantly upregulated in KL35. qRT-PCR results showed that the relative expression level of the gene encoding 4CL (*Os02g0697400*) was significantly higher in KL35 compared to TN1 under non-feeding conditions. After feeding, the expression of this gene showed a downward trend in both resistant and susceptible varieties, and no significant difference was observed between the two after 48 h. Additionally, after 48 h of feeding, the gene encoding C4H (*Os05g0320700*) was significantly upregulated in KL35, while its expression was not significantly altered in TN1 (**Figure 8B**).

### 3.6 Effects of exogenous CGA on WBPH bioassays

**Figure 9:**
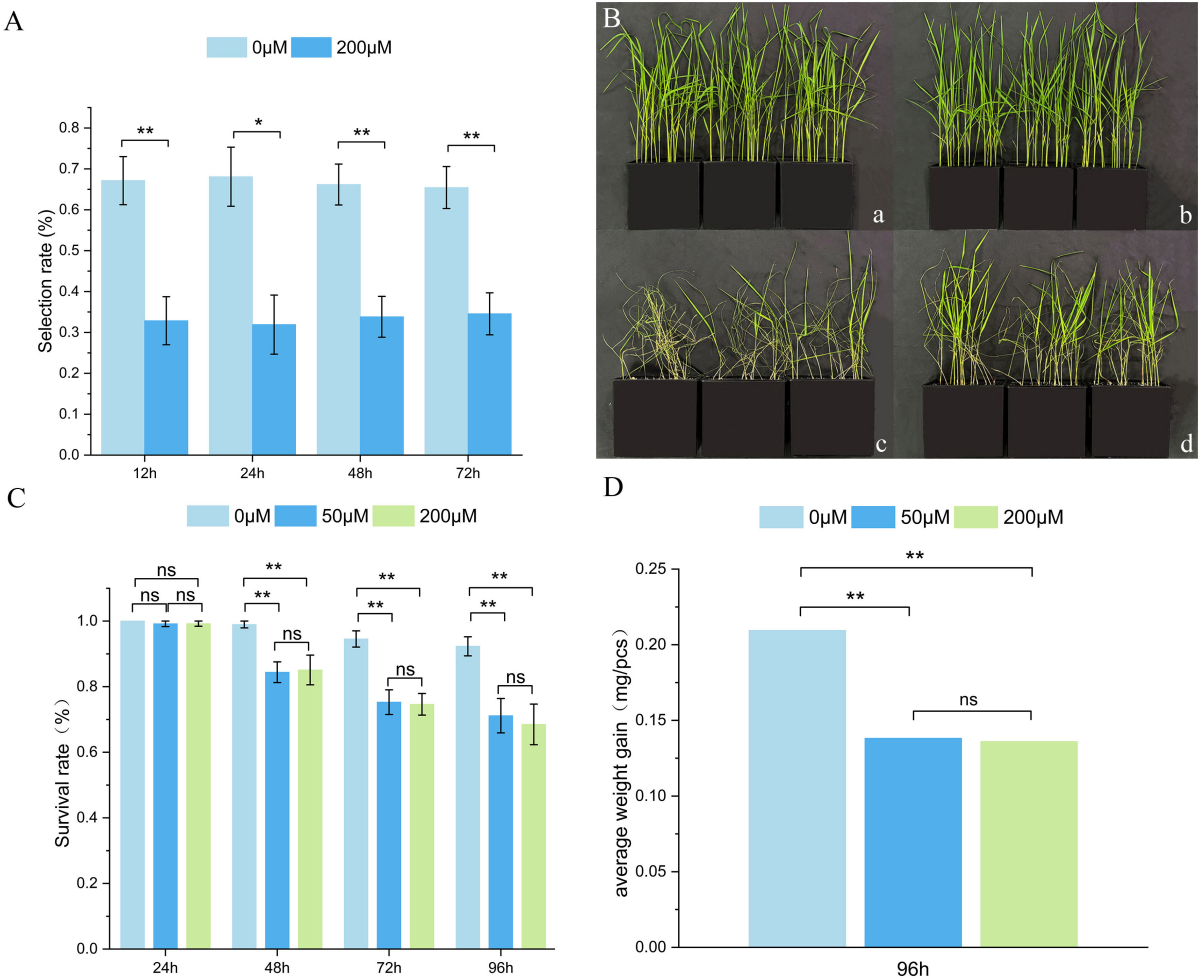
Effect of exogenous CGA by WBPH bioassay on TN1. **(A)** WBPH nymphs selection profiles from CGA application across time; **(B)** Phenotypic effect of seedling sprayed without/with 200µM CGA. (a, c) Plants sprayed with water before (a) and after (c) WBPH feeding; (b, d) plants sprayed with CGA before (b) and after **(d)** WBPH feeding; **(C)** Effect of CGA concentration by nymphs survival rate across 4 time points; **(D)** Effect of CGA concentration by nymphs dry weight gain at 96h after application. **, *, and ns indicate significance levels of p< 0.01, 0.05, and no significance, respectively, with independent samples t-test.

The selection rate of WBPH nymphs on TN1 treated with 200 μM CGA was significantly lower than that on TN1 treated with distilled water, but the selection rate did not change significantly over time, indicating that CGA has a strong antixenosis effect on WBPH (**Figure 9A**). As shown in **Figure 9B** and **Table 4**, after WBPH feeding, TN1 plants treated with distilled water suffered the most severe damage, with the greatest weight loss, and their dry weight was significantly lower than that of TN1 plants treated with 200 μM CGA. Additionally, the FPLI of TN1 treated with distilled water was 100, which was significantly higher than the FPLI of 80.95 for TN1 treated with 200 μM CGA. This indicated that CGA improved the tolerance of TN1 to WBPH damage.

**Table 4:** Evaluation of tolerance to WBPH infestation among TN1 treated with CGA.

Varieties	Total dry weight (g) of uninfected plants	Total dry weight (g) of infested plants	The average resistance level at sampling time	Functional plant loss index (FPLI)
TN1	0.42 ± 0.012^ns^	0.20 ± 0.004	9 ± 0.000^*^	100 ± 0.000^*^
TN1 + 200 μM CGA	0.41 ± 0.014	0.24 ± 0.003^***^	6 ± 0.577	80.95 ± 3.090

***, *, and ns indicate a significant difference (*p*< 0.001), a significant difference (*p*< 0.05), and no significant difference (*p* > 0.05) determined by independent samples’ t-test.

After feeding on rice treated with 50 μM and 200 μM CGA for 48 h, the survival rate of 2nd-3rd instar WBPH nymphs was significantly reduced compared to the control group, with the survival rate showing a declining trend as feeding time increased. However, no significant difference in survival rate was observed between the 50 μM and 200 μM CGA treatment groups (**Figure 9C**). Since no significant reduction in survival rate occurred after feeding on CGA-treated rice for 24 h, it is speculated that CGA does not cause immediate death in WBPH nymphs but instead affects their growth, development, or feeding behavior, leading to death over time. After feeding on rice treated with 50 μM and 200 μM CGA for 96 h, the average dry weight of WBPH nymphs was significantly lower than that of the control group, but no significant difference in dry weight was observed between the 50 μM and 200 μM treatment groups (**Figure 9D**).

## 4 Discussion

Throughout their long-term co-evolution with insects, plants have developed complex defense systems to counter insect herbivory through various morphological and biochemical mechanisms (War et al., 2012). Pest resistance is generally achieved through one or a combination of the following three defenses: antixenosis, which prevents migration or oviposition by driving away or repelling insects; antibiosis, which reduces insect survival, growth, or reproduction after ingestion of tissues; and increased tolerance (Yang et al., 2020). In this study, the resistant rice variety KL35 demonstrated robust resistance to the WBPH through a combination of these mechanisms. Specifically, KL35 exhibited strong antixenosis by repelling both nymphs and adults of WBPH, as well as significant tolerance to WBPH damage, enabling it to compensate for the inflicted harm. Additionally, KL35 displayed notable antibiosis effects, primarily by inhibiting the growth, development, and reproduction of WBPH. In contrast, the resistant varieties, 79–1163 was superior in antibiosis but showed poor tolerance and antixenosis; G577 showed outstanding antixenosis; BG276–6 and G577 exhibited high levels of tolerance (Huang et al., 2019). The genetic basis for WBPH resistance would differ with different varieties, which merits attention in WBPH resistance breeding practice.

Also, resistance to WBPH can differ in different rice varieties with diverse defense mechanisms at different growth stages. For instance, IR54751 displayed high resistance to WBPH at both seedling and tillering stages (Fan et al., 2018), whereas KL35 confers robust resistance at the two-leaf stage, and is largely related to the production of volatile compounds. It is also inducible that the plant tissue structure and physicochemical properties at different development stages can be involved in WBPH resistance, but further research is needed at molecular, biochemical, and genetic levels.

Metabolic analysis of rice plants subjected to WBPH feeding revealed the resistance of KL35 involves several biochemistry pathways including the biosynthesis of flavonoids and plant hormone signal transduction, which is consistent with the findings in BPH studies (Zhang et al., 2022; Shi et al., 2023). With the dynamic change of the complex metabolites in KL35 after WBPH feeding, a key metabolite CGA was identified within the flavonoid biosynthesis pathways as a significant marker, which suggests CGA may play a pivotal role in KL35 resistance to WBPH. The postulation is based on the following two observations. First, CGA is a well-known phenolic compound associated with antioxidant activity and insect resistance in other plant species (Lee et al., 2017; Liu et al., 2017), especially with a high level of CGA Chrysanthemum (*Dendranthema grandiflora*) is more resistant to *Frankliniella occidentalis* (Leiss et al., 2009). Secondly, among different secondary metabolites, CGA stands out in the defensive response of rice resistance to BPH during the infestation process (Uawisetwathana et al., 2019). In this study, higher CGA content in KL35, even before insect feeding, suggests a possible constitutive involvement of this compound in KL35’s resistance.

CGA has been shown to be an effective insecticidal compound, that impairs different biological functions of the herbivorous insects (Kundu and Vadassery, 2019; Lin et al., 2023). Mechanisms for CGA on insect resistance have been postulated. First, CGA is involved in plant defense as an antifeedant or toxin that directly deters or impairs WBPH feeding (War et al., 2012; Asif et al., 2022). Second, CGA may contribute to changes in plant physical and chemical traits, such as cell wall thickening or the accumulation of defensive compounds, which make it more difficult for WBPH to access phloem sap (Li et al., 2018; Sun et al., 2024). Additionally, when ingested by WBPH through plant sap, CGA may directly interfere with their physiological processes, such as digestion, development, and reproduction (Zhang et al., 2013; Sripontan and Hwang, 2016).

To address the role of CGA on WBPH resistance in KL35, the integrated analysis of metabolomics and transcriptomics revealed three regulatory genes C4H (*Os05g0320700*), 4CL (*Os02g0697400*), and HCT (*novel.1556*) were found to be significantly upregulated in KL35. These genes are exactly located in two of the three biosynthetic routes of CGA biosynthetic pathways (Kundu and Vadassery, 2019), so that the resistance of KL35 can be partially attributed to the increased CGA biosynthesis. However, the increased CGA may be the consequence of some other mechanisms that have been triggered by WBPH feeding. When an insect feeds on a plant it causes a mechanical wound and introduces elicitors from insect saliva into plant tissue. These wounds and elicitors trigger a cascade of defense signaling pathways including jasmonic acid (SA), salicylic acid (SA), and ethylene (ET) pathways. Also, plant activates the phenylpropanoid pathway for phenolic biosynthesis. Details of the interaction between insect elicitors from WBPH and the initialization of the pathways in the host plant, especially the important genes, should be among the future research challenges.

Under the non-feeding experiment, KL35 also showed a higher level of CGA than TN1, which indicates a likely elevated constitutional expression of the gene(s) in the pathway including the regulatory factors. With the available whole genome of TN1 (Panibe et al., 2021), the detailed differentiation between the two lines may be revealed from the genomic level when other factors are clarified. Analysis of 5 copies of the 4CL gene in the rice genome differentiates the roles of the isoforms of this gene for flavonoid biosynthesis and lignin synthesis (Sun et al., 2013). Breeding for WBPH-resistant variety can be achieved by introgression of the corresponding gene(s) through backcross such as marker-assisted selection.

In this study, we observed a significant increase in JA content in KL35 following WBPH feeding, accompanied by a corresponding rise in CGA levels, indicating that JA responds to the stress induced by WBPH. This finding aligns with previous studies demonstrating that JA acts as a key signaling molecule in response to biotic stress, including herbivory, and plays a regulatory role in the biosynthesis of secondary metabolites such as CGA (Ruan et al., 2019; Liu et al., 2024). JA and its derivative, methyl jasmonate (MeJA), are well-known for their roles in mediating plant defense responses, including the activation of metabolic pathways involved in the production of defensive compounds like CGA. The observed increase in CGA content in KL35 after WBPH feeding is consistent with studies showing that MeJA treatment can enhance CGA accumulation in various plant species. For example, in *Gardenia jasminoides* cells, MeJA treatment significantly promoted the accumulation of CGA and its derivatives, such as caffeoylquinic acids (CQAs) (Liu et al., 2021). Similarly, in *Physalis angulata* L., MeJA treatment upregulated the transcription levels of genes involved in CGA biosynthesis, leading to a significant increase in CGA content (Zhan et al., 2020). Based on these findings, we speculate that JA may regulate CGA synthesis, working together to defend against WBPH. However, unlike previous studies that used exogenous MeJA application to confirm the relationship between JA signaling and CGA accumulation, we did not experimentally manipulate JA levels in KL35 to directly test this hypothesis. Therefore, while our results strongly suggest that JA regulates CGA synthesis in response to WBPH infestation, further research involving exogenous JA application or genetic manipulation of JA biosynthesis is needed to establish a definitive causal relationship.

In summary, the mechanisms for the resistance of KL35 to WBPH were evaluated in this study through integrated metabolomic and transcriptomic analyses, and CGA was identified as a prominent marker compound for WBPH resistance. Two genes C4H (*Os05g0320700*) and 4CL (*Os02g0697400*), were identified and postulated as key players in the CGA biosynthesis pathway in KL35. As a plant-derived and environment-friendly compound, CGA could be a potentially important compound for rice WBPH resistance agriculture. Through agronomy management by intercropping with CGA-rich plants, and by direct application of this plant-derived biopesticide, CGA can offer an eco-friendly alternative for rice WBPH management. Genetic engineering and molecular breeding with the targeted key genes to enhance the CGA level will provide new improvements in rice WBPH resistance.

## Data Availability

The original contributions presented in the study are publicly available. This data can be found here: NCBI, SAMN48358999, SAMN48359000, SAMN48359001, SAMN48359002.
